# Teledentistry as a Supportive Tool for Dentists in Pakistan

**DOI:** 10.1155/2021/8757859

**Published:** 2021-09-08

**Authors:** Rootab Subhan, Waleed A. Ismail, Sadia Musharraf, Mylla Khan, Resham Hafeez, Mohammad Khursheed Alam

**Affiliations:** ^1^Department of Periodontics, Shifa College of Dentistry, Shifa Tameer-e-Millat University, Islamabad 44000, Pakistan; ^2^Periodontics Unit, School of Dental Sciences, Health Campus, Universiti Sains Malaysia, 16150 Kubang Kerian, Kota Bharu, Kelantan, Malaysia; ^3^Department of Periodontology, Army Medical College, National University of Medical Sciences, Rawalpindi 46000, Pakistan; ^4^Department of Preventive Dentistry, College of Dentistry, Jouf University, Sakaka, Al Jouf 72345, Saudi Arabia

## Abstract

The current scenario of the COVID-19 pandemic has forced dentists to seek different options for delivering healthcare services other than the in-person direct examination in clinical practice. Teledentistry is one of the options for remote patient care and monitoring. *Objective*. The present survey was conducted to assess the knowledge and perception of the dentists in Pakistan regarding teledentistry as an emergent supportive tool. *Materials and Methods*. A self-administered, close-ended, and prevalidated survey questionnaire was used, comprising 21 questions, and distributed electronically via e-mail, WhatsApp, and Facebook Messenger to evaluate the knowledge and perception of dentists regarding teledentistry. The data collected was compiled in a systematic manner and analyzed in terms of frequency (yes/no). *Results*. Out of a total of 350 dentists, 325 responded to the questionnaire, and it was seen that 62.5% of them did not have knowledge about teledentistry prior to COVID-19. 65.8% of dentists considered the practice of teledentistry in nonpandemic situations in the future. *Conclusion*. In the present study, it was observed that most of the dental professionals had inadequate knowledge about teledentistry before COVID-19, but the awareness and perception regarding teledentistry were currently satisfactory among the dental professionals in Pakistan. This emerging trend gives a positive hope for the implementation of teledentistry in the healthcare setup of Pakistan in the near future, as it will prove to be beneficial for safe dental practice during times of pandemic and even after.

## 1. Introduction

At the beginning of the year 2020, a pandemic emerged affecting the entire world. The healthcare system has been critically affected with the rise of COVID-19, which has created a global healthcare concern that greatly halted the normal healthy lifestyle of the patients as well as dentists. Dental treatment invariantly involves close inspection, examination, diagnostic, and therapeutic interventions of the patient's oronasal-pharyngeal region. Therefore, the dental professionals are at high risk of acquiring infection with COVID-19 [1]. During the COVID-19 pandemic, routine dental treatments have been interrupted, and only critical dental procedures and surgeries are being performed.

However, looking at the widespread situation created by COVID-19, this seems that it will not end anytime soon. In fact, the WHO feared that the COVID-19 virus will never go away and will become endemic in our communities [2]. Along with all other health issues, dental practice is also affected, so there is a need of restructuring and introducing new modernized electronic methods of healthcare delivery to pursue dental care with minimal risk of transmission of disease and cross-infection. In addition to these, the primary concern of dentists during the pandemic is the infection control practices and the demand to maintain standard precautions in the care of dental patients, during and even after the period of this pandemic [3].

The term “telehealth” is given to public health and healthcare services, conveyed with the aid of information technology and communication to provide healthcare at a site distant to the patient to facilitate patient consultation, diagnosis, self-care, and treatment planning. Teledentistry is a subtitle of telehealth which facilitates dental care at a distance with the aid of information technology and communication. Legal and professional obligations in all dental practices that apply to in-clinic care also apply to teledentistry [4].

In addition, dental care is being transformed gradually due to the opportunities provided by the technology. Teledentistry is progressing rapidly with the increased incidence of COVID-19 [5]. In 1997, Cook defined teledentistry as “the practice of using video-conferencing technologies to diagnose and provide advice about dental treatment over a distance” (see [6]).

Teledentistry is not only based on telecommunication but also involves the exchange of clinical information and data. Also, it is important to understand how dentists perceive teledentistry as a supportive tool during the pandemic crisis and how this may affect their future practice of teledentistry, particularly with infection control.

Teledentistry in the current scenario of COVID-19 focuses on “dental triage,” the relief of pain or infection, provision of dental care by remote consultation, planning, and scheduling of definitive dental treatment. Technological advances have the potential to improve the current scenario of routine dental practice. It has a variety of uses that include the following:

1. Help improve oral healthcare access to the patients
2. Improve the oral healthcare delivery
3. Help for under-served populations
4. Increase specialist availability
5. Economic and time saving

Teledentistry as a method of diagnosis and treatment planning includes all branches of dentistry [7]. Berndt et al. carried on a survey on unprivileged and deprived children, wherein orthodontic treatment was being surveilled from a distance with optimistic results using teledentistry [8]. Another study by Park et al. specified that the advancements and availability of information technology could lead to the use of telemedicine in oral and maxillofacial surgery [9]. Lienert et al. stated that telemedical services were found to be beneficial for dental trauma-related cases in a Swiss telemedical center and proved to be valuable where a specialty dentist was not available [10]. Snow et al. observed that teledentistry permitted distant, cost-effective, and specialist dental consultations for rural Australians [11]. If the projections on the shortages of dentists in the next decade come to pass, teledentistry will be important not only for rural areas but also for our urban and suburban populations [12]. Interprofessional communications will improve dentistry.

However, the research conducted in Pakistan on teledentistry is limited among the dentists for the evaluation of knowledge and awareness regarding teledentistry. Hence, the present study was conducted among the dentists to assess their knowledge and perception regarding teledentistry, provided as a supportive tool for patients' healthcare needs.

## 2. Materials and Methods

### 2.1. Study Design and Population

The present study was conducted in a time span of 4 months, between September 2020 and December 2020. The study population comprised 350 dentists from all the 9 specialties of dentistry currently working in Pakistan. Participants of the study included dentists with postgraduate qualifications, consultants and specialists, undergraduate dentists that included trainees and demonstrators, general dentists, and interns from private and government sectors of Pakistan. The respondents were chosen through nonprobability purposive sampling.

### 2.2. Ethical Approval

As this was a questionnaire-based awareness and perception study, an exemption for ethical approval was taken from the Institutional Review Board. A written informed consent was obtained from all the dentists to whom the questionnaire was circulated.

### 2.3. Data Collection and Analysis

A self-administered, close-ended, and prevalidated questionnaire, consisting of 21 questions to inquire about dentists' sociodemographic information, knowledge, and perception of dentists regarding teledentistry, was administered [13]. The questionnaire was distributed by a single investigator. The investigator sent the questionnaire using e-mail, WhatsApp, and Facebook Messenger, which was to be filled and submitted online. The questionnaire comprised three parts which included the following:

- *Part 1*: sociodemographic details
- *Part 2*: questions relating to knowledge regarding teledentistry use during COVID-19
- *Part 3*: questions relating to perception of dentists regarding teledentistry as a supportive tool during COVID-19

The data was collected, compiled, arranged in a systematic manner, and analyzed in terms of frequencies (yes/no) using SPSS Version 26.

### 2.4. Inclusion and Exclusion Criteria

Inclusion criteria for this study were as follows:

- Registered dentists with at least a Bachelor of Dental Surgery degree, practicing in Pakistan
- Dentists involved in direct dental care of patients

Exclusion criteria for this study were as follows:

- Dentists not registered with the Pakistan Medical Commission
- Dentists unwilling to participate in the survey
- Dentists not responding on the predecided dates (September–December 2020) of the study

## 3. Results

The questionnaire was circulated amongst a total of 350 dentists from all the specialties of dentistry in Pakistan, out of which 325 dentists responded and gave their consent to participate in the survey. The response rate was 92.8%.

Demographically, the ratio of male-to-female respondents was almost equal (53.5% and 46.5%, respectively). Majority of the respondents were from the age group of 25-34 years old (55.1%), with the 18-24-year-old bracket coming in second (26.2%). Therefore, a vast majority of this study's population was based on the younger lot (Table 1).

It was observed that 62.5% of the dentists did not have knowledge about teledentistry prior to COVID-19, but currently, the majority of them know about teledentistry (68.6%). The general awareness regarding teledentistry was satisfactory among the participants, as can be seen in the response rates in Table 2.

89.2% of dentists believe teledentistry could be useful in facilitating access to oral healthcare during the pandemic, but 65.8% of dentists agreed to practice teledentistry in a nonpandemic situation as well. A relatively high response rate was achieved for whether teledentistry can be added to routine dental practice, with 68.9% of dentists answering in an affirmative.

About 85.8% of the dentists would recommend the government to take the initiative whereby patients could obtain treatment needs from a central facility connected via teledentistry, but at the same time, 47.4% of dentists considered teledentistry not to be a convenient and supportive tool in Pakistan due to the country's major challenges of illiteracy, population below the poverty line, and lack of infrastructure.

## 4. Discussion

The present cross-sectional study unveiled a topic that earned considerable interest in relation to dynamic advancement in dentistry (teledentistry) among dentists in Pakistan during COVID-19. Teledentistry is not a notion new to mankind and has been developing since 1994, but there have been major new advancements over the years in the field of dentistry. Delivering oral healthcare to the patients has dramatically been influenced during the pandemic, with dentistry considered as a very risky profession in present circumstances. Droplets and aerosols generated during dental procedures, being the main source of transmission, endanger the dental practitioners. Teledentistry during the time of COVID-19 is the only reliable way to provide consultation and treatment using technology [14]. Teledentistry allows long-distance communication by avoiding person-to-person contact, observing social distancing, permitting exchange of clinical information and images, and facilitating remote dental care, patients' education, and guidance which is recommended by healthcare authorities across the world.

The current literature highlights the significance of teledentistry. Teledentistry has numerous subdivisions such as teleconsultation, telediagnosis, telemonitoring, and teletriage, each having important functions relevant to dental practice. Teleconsultation assists in reducing noncritical patient referrals, thus lowering the burden on already strained healthcare systems. Telediagnosis helps in the use of technology to exchange patient records, intraoral images, and radiographic images to diagnose oral diseases remotely [15]. Teletriage emphasizes on patients requiring urgent dental care following remote evaluation of oral health, minimizing the need of nonessential travel, keeping in view the pandemic situation [16].

Teledentistry can benefit both the dentists and patients, but somehow, insufficient data is available concerning dentists' perception about adopting it in Pakistan. This was the main purpose of our study, and the general observation arising from our data is that dentists appreciate teledentistry for its capability to improve patient care and enhance oral health practices, which is unanimous to a similar survey conducted in Canada, where the majority of the dentists were in support of the use of teledentistry in better healthcare delivery [17]. Our results showed that a very small percentage (37.5%) of the dentists had knowledge about teledentistry before COVID-19, but a vast majority (86.8%) were aware that it is the practice of use of computers, internet, and technologies to diagnose and provide advice over a distance. Similar results were seen in surveys conducted in the US, India, and Rwanda, where awareness about teledentistry among dental professionals was very high [18–21].

The role of teledentistry has also been seen and assessed in dental education [8, 22]. In this study, almost 89.5% of dentists agreed on upgradation and implementation of health education by teledentistry. A survey about teledentistry in India observed that 58% of dentists agreed with the statement that teledentistry is good for dental education and training over internet [23]. A study carried out in 2015 stated that 50% of the practicing dentists agreed on the execution of health education by teledentistry [5]. Two main categories of health education by teledentistry are self-instruction and video conferencing. Latif et al. reported in a study in 2016 that the last seminar conducted by the E-Health Association of Pakistan was in 2011, and to date, no further advances have been made in this field [24].

In our study, 63.4% of dentists believe that teledentistry helps in reducing costs for dental practices. 59.7% of respondents in a study carried out in India agreed teledentistry reduces costs for dental practice [13]. A study conducted by Bauer and Brown also showed similar results [25]. Theoretically, it is logical to think that by assessing the patients over-the-air, not only will the dental costs be minimized but it would be cost-effective and a comfortable way for the patients as well.

About 87.1% of the dentists agreed on consulting or communicating with specialist dental professionals through the internet or mobile phones in this survey. This allows gathering and exchanging informative data with a dental specialist who may have more expertise in a particular area. This data can be used to support the delivery of dental care, diagnosis consultation, and treatment. In comparison to a study on postgraduate dental students in Kanpur, India, only 58.4% of respondents agreed on this point [26]. This will specially be useful for dental professionals in remote areas, who will be able to communicate with specialists or more skilled dental practitioners about specific patients' problems using teledentistry.

In our survey, more than half of the dentists (57.5%) had this perception that teledentistry can be applied in any branch of dentistry. A similar study done in Pakistan depicted that 35.9% of dental professionals held the same perception [9]. Another similar study performed in India recorded that 42% of dentists agreed teledentistry can be applied in any branch of dentistry [6]. Useful applications of teledentistry in various branches of dentistry were observed in a literature review which indicated that benefits can be gained in the fields of Oral Medicine and Diagnosis, Oral and Maxillofacial Surgery, Endodontics, Orthodontics, Prosthodontics, Periodontics, Pediatric, and Preventive Dentistry [4]. Bradley et al. also mentioned the benefits of teledentistry especially in the branch of Oral Medicine [27]. Teledentistry proved to be useful in Endodontics for the diagnosis of periapical lesions as well [28]. In Prosthodontics, diagnosis with treatment planning has been performed by video conferencing [29]. Applications of teledentistry in Orthodontics have also been mentioned in a prior study [8].

According to the present study, awareness regarding teledentistry is high among undergraduate dental professionals as compared to general dentists, interns, and postgraduates, which is similar to a study that stated less awareness among general dentists about teledentistry in comparison to postgraduates and undergraduates [30]. This can be attributed to the fact that the modern curricula at the undergraduate dentistry level is based on newly discovered technology as compared to what was being taught almost half a decade back. Also, the new generation is more inclined towards technology themselves; thus, their growing interest in teledentistry does not come as a surprise.

It was seen that 65.8% of dental professionals believe that teledentistry can offer novel solutions to resume regular dental practice during nonpandemic situations. Teledentistry will prove to be useful for future assistance in planning appointments whereby new patients can be examined via two methods. The initial stage will involve history taking and consent, and a second stage will include face-to-face visit whereby examination, diagnosis, and treatment can be completed in one visit. The number of in-patient appointments would reduce in hospitals/clinics, thus limiting the spread of COVID-19.

85.8% of dentists suggested that the government of Pakistan should take the initiative that would help patients obtain advice on treatment needs from a central facility connected via teledentistry during COVID-19 and in nonpandemic situations too. Pakistan, still being a developing country, lags behind in technology as compared to other developing countries of the South Asia region. Therefore, a drastic advancement needs to be done in this regard for teledentistry to flourish in the country, and this change will only be brought about if proper steps are initiated at a governmental level in provinces.

One integral aspect to consider here is that dentists, who are involved in teledentistry, must put every effort to ensure the security of their systems and gather data as well as processing of all types of collected data. For example, data encryption, password protection, and user access logs can help in preventing misuse of information of most of the people and ultimately protecting patient confidentiality. Dentists are encouraged to remain aware and be vigilant in terms of the legal needs in their states of practice.

## 5. Conclusion

This study shows that despite most of the participants having inadequate knowledge about the term “teledentistry” before COVID-19, they expressed positive prospects regarding its use. However, the routine practice is still low in Pakistan due to barriers such as unavailability of advanced technology and lack of education and training of dental professionals. There is a need to enhance the knowledge regarding teledentistry and promote its utilization in order to triage patients in this time of COVID-19, and even after. To ensure implementation of teledentistry, designing new policies and dental education programs is mandatory. Teledentistry may continue to be used after the pandemic as a means of consultation and provide easy access to dental professionals and patients.

## Figures and Tables

**Table 1:** Sociodemographic characteristics.

Sample characteristics	Frequency (%)
Age (years)	
18-24	85 (26.2)
25-34	179 (55.1)
35-44	31 (9.5)
45-54	16 (4.9)
55-64	9 (2.8)
>65	5 (1.5)
Gender	
Male	174 (53.5)
Female	151 (46.5)

**Table 2:** Responses in knowledge, awareness, and attitude-related questions.

	Knowledge, awareness, and attitude-related questions	Yes (%)	No (%)
Q1	Have you heard about teledentistry before COVID-19?	37.5	62.5
Q2	Do you know what teledentistry is?	68.6	31.4
Q3	Do you think teledentistry is about the practice of use of computers, internet, and technologies to diagnose and provide advice over a distance?	86.8	13.2
Q4	Can teledentistry help to consult with an expert about a specific patient's problem?	87.1	12.9
Q5	Can teledentistry help to monitor the patient's oral health in order to avoid dental visits?	71.1	28.9
Q6	Do you think that teledentistry should be a part of dental education training of dentists?	89.5	10.5
Q7	Can teledentistry facilitate the access of oral healthcare during a pandemic situation?	89.2	10.8
Q8	Do you think that teledentistry can be a good tool for oral hygiene training?	82.8	17.2
Q9	Do you think teledentistry is a convenient form of oral healthcare for both the dentist and patients?	70.2	29.8
Q10	Do you think that patients can be examined accurately via computers and intraoral cameras as in the traditional office setting?	33.2	66.8
Q11	Can teledentistry be applied in any branch of dentistry?	57.5	42.5
Q12	Can teledentistry be added to routine dental practice?	68.9	31.1
Q13	Can teledentistry help in reducing costs for dental practices?	63.4	36.6
Q14	Do you think that teledentistry saves time for the dentist?	68.3	31.7
Q15	In Pakistan, can teledentistry be successful as a supportive tool with major challenges of illiteracy, population below the poverty line, and lack of infrastructure?	52.6	47.4
Q16	Do you think that teledentistry can increase accessibility of the specialists to rural and undeserved communities for their dental needs?	69.5	30.5
Q17	Should teledentistry be a regular practice in nonpandemic situations?	65.8	34.2
Q18	Will you recommend that government should take an initiative whereby patients could obtain advice on treatment need from a central facility connected via teledentistry?	85.8	14.2

## Data Availability

The raw data used to support the findings of this study are included within the article.
